# Ultra-compact and high-precision differential detection method based on liquid crystal polarization grating for miniature atomic magnetometer

**DOI:** 10.1515/nanoph-2024-0309

**Published:** 2024-10-04

**Authors:** Zhibo Cui, Yuhao Wang, Ying Liu, Mingke Jin, Jie Sun, Yueyang Zhai, Xiangyang Zhou, Zhen Chai

**Affiliations:** Key Laboratory of Ultra-Weak Magnetic Field Measurement Technology, Ministry of Education, School of Instrumentation and Optoelectronic Engineering, 12633 Beihang University, Beijing 100191, China; Institute of Large-Scale Scientific Facility and Centre for Zero Magnetic Field Science, 12633 Beihang University, Beijing 100191, China; Hangzhou Extremely Weak Magnetic Field Major Science and Technology Infrastructure Research Institute, Hangzhou 310051, China; Beihang Hangzhou Innovation Institute, Hangzhou 310052, China; Hefei National Laboratory, Hefei 230088, China

**Keywords:** liquid crystal polarization grating (LCPG), optical rotation angle, differential detection, atomic magnetometers (AMs)

## Abstract

Atomic magnetometers (AMs) that use alkali vapors, such as rubidium, are among the most sensitive sensors for magnetic field measurement. They commonly use polarization differential detection to mitigate common-mode noise. Nevertheless, traditional differential detection optics, including polarization beam splitters (PBS) and half-wave plates, are typically bulky and large, which restricts further reductions in sensor dimensions. In this study, a combination of liquid crystal polarization grating (LCPG) and liquid crystal quarter-wave plate is used for differential detection in AMs, with magnetic field strength determined by measuring the intensity of two diffracted beams from the LCPG. The experimental findings indicate that the fabricated LCPG exhibits a circularly polarized extinction ratio of 3,656 and achieves an average diffraction efficiency of 99 %. In addition, the differential detection method based on LCPG can achieve an angular resolution of 1.48 × 10^−7^ rad. Subsequently, the method is employed in an AM to achieve an average magnetic sensitivity of 13.8 fT/Hz^1/2^. Compared to the PBS-based differential detection method, this method enhances the magnetometer response coefficient by 13 % and achieves co-side distribution of the two diffracted beams, thereby avoiding the need for additional vertical optical paths. The effective thickness of the detection optics is reduced to the micrometer scale, allowing for future integration as thin films onto microfabricated vapor cells. This study offers a practical solution for miniaturized AMs with exceptionally high sensitivity.

## 1 Introduction

In recent decades, the performance of atomic magnetometers (AMs) has greatly improved, attaining sensitivities in the sub-fT/Hz^1/2^ range [1], [2]. The diverse applications of highly sensitive AMs, such as geographic measurements [3], fundamental physics testing [4], and navigation [5], drive a keen interest in their development. AMs are expected to replace superconducting quantum interference devices, particularly in bio-magnetic imaging including magnetoencephalography [6], [7] and magnetocardiography [8], [9]. In response to the need for enhanced resolution in biomagnetic measurements, future developments in AMs will focus on achieving smaller size, reduced costs, and increased sensitivity [10], [11].

In general, high-sensitivity AMs use a circularly polarized beam to excite alkali atoms, such as rubidium. Then, a nonresonant, linearly polarized beam is used to detect the precession of atomic spins in the presence of a magnetic field [12]. Typically, the rotation angle of the polarization plane (namely, optical rotation angle) reflects this precession before and after the probe beam traverses the vapor cell. Numerous methods exist for detecting the optical rotation angle, including polarization differential detection (PDD), Faraday modulation, and photoelastic modulation [13], [14]. The PDD method is the most widely used among these techniques in AMs because it offers a simple optical path structure and effectively reduces common-mode noise caused by laser intensity fluctuations. Employing microfabricated vapor cells and vertical-cavity surface-emitting lasers has facilitated the partial miniaturization of AMs [15], [16]. However, the refractive optical elements used in the PDD method, such as polarization beam splitters (PBS) and half-wave plates (HWP), still hinder the integration of AMs. Typically, these refractive optical elements achieve the desired phase modulation through phase accumulation along the light propagation. For example, a standard PBS is usually made by joining two birefringent crystals with orthogonal optical axes. At the contact surface, the incident beam is both refracted and reflected, resulting in two linearly polarized beams oriented perpendicular to each other. Consequently, the PBS thickness must be greater than the diameter of the spot. Therefore, it is difficult to fabricate a compact, low-cost, and lightweight device using conventional refractive optics, and a large volume of the detection system are inevitable. As chipization advances, traditional refractive optics will give way to novel compact and ultra-thin devices. Furthermore, the precision of PDD relies on the performance of polarized optics, necessitating a high-performance integrated device to substitute the traditional polarized optics.

Liquid crystals (LCs), known for their outstanding light transmittance, high optical birefringence, and continuous gradient pointing vector distribution, can modulate the amplitude, phase, and polarization of electromagnetic waves [17], [18], [19]. In recent years, planar optical elements based on LCs have drawn considerable attention benefitting from the Pancharatnam–Berry (PB) geometric phase control [20–24] . Distinct from the conventional phase accumulation, the LC optics based on the PB phase introduce abrupt phase change by rotating the anisotropic LC molecules. Therefore, LC optics offer a considerable reduction in size compared with traditional refractive optics. Liquid crystal polarization gratings (LCPGs), as a standard LC optics, demonstrate outstanding polarization sorting performance. For instance, the LCPG can divide elliptically polarized light into two orthogonal beams: right-handed circularly polarized (RCP) and left-handed circularly polarized (LCP). The intensity of each beam depends on the ellipticity of the incident light. Researchers have developed the method using LCPG and quarter-wave plate (QWP) to measure optical rotation [25], [26]. Recent studies have achieved optical rotation detection precision of up to 0.002° (3.5 × 10^−5^ rad) with this method [27], validating its high-precision detection capability. Nevertheless, previous studies predominantly concentrated on the utilization of this method for measuring the concentration of liquid, thereby limiting its application. Here, for the first time, we contemplate its application in the domain of atomic sensor rotation angle detection. Considering the miniature AMs’ ability to detect incredibly faint magnetic fields in the order of femtosla or sub-femtosla, an angular detection resolution ranging from 10^−7^ to 10^−8^ rad is necessary [28]. Hence, there is a need for additional enhancements to the LCPG to fulfill the requirement for high-precision detection of AMs. Advancements in LC fabrication technology will enable further improvements in the performance of LC optics. This technology is expected to replace the conventional refractive optics used in atomic sensors [29], enhancing sensor performance and reducing sensor size.

**Figure 1:**
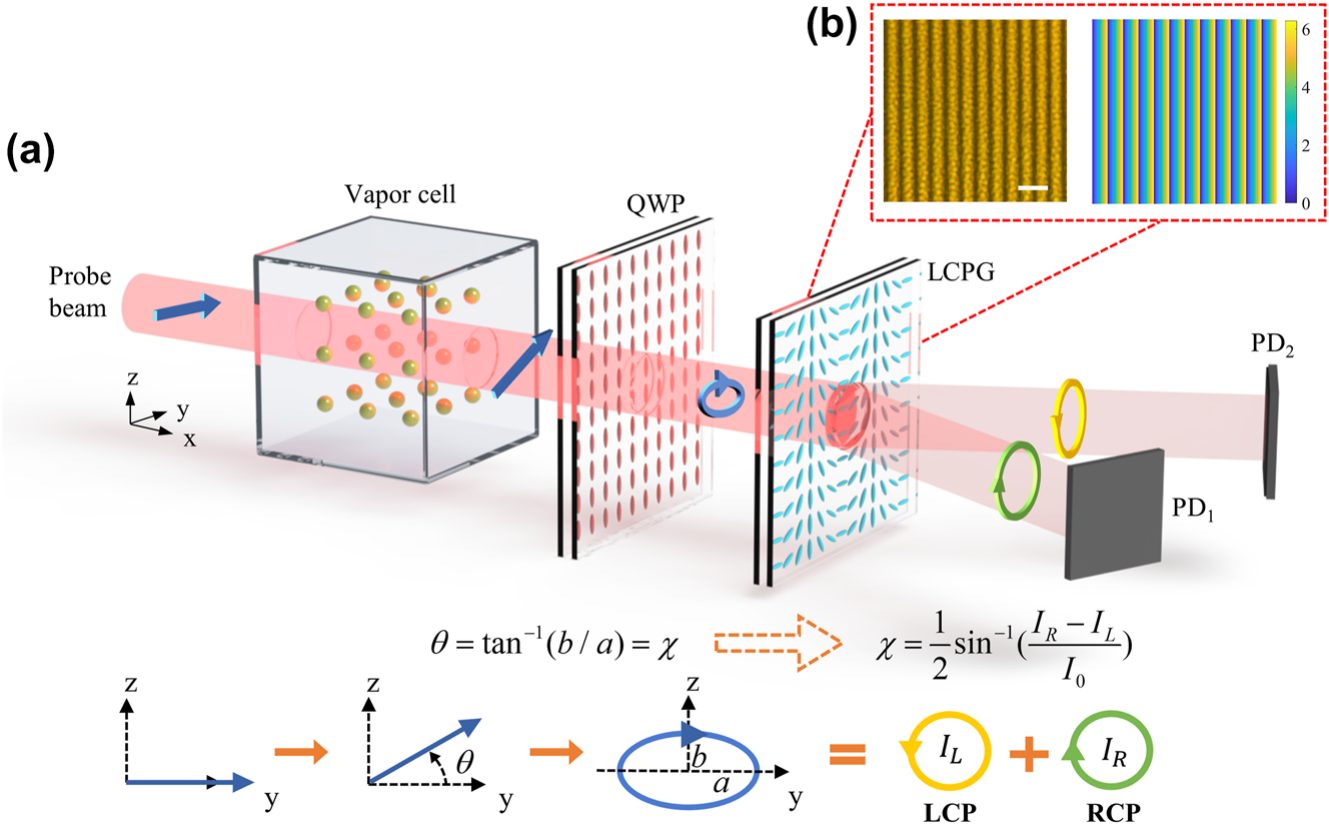
The principle of differential detection method based on LCPG. (a) Schematic illustrating the LCPG-based differential detection principle. The equations represent the conversion of the optical rotation angle *θ* to the ellipticity angle *χ*, which corresponds to the polarization state evolution process. The *θ* can be determined by detecting the intensity of RCP and LCP. QWP: quarter-wave plate; LCPG: liquid crystal polarization grating; PD: photodetector; LCP: left circularly polarized; RCP: right circularly polarized. (b) Optical micrographs of LCPG measured at 100× magnification polarizers, scale bar: 20 μm, and the phase distribution of LCPG, size 110 × 110 μm^^2^.

This study developed an efficient and compact differential detection method for detecting atomic spin precession by employing an LCPG and a liquid crystal QWP. As illustrated in Figure 1a, linearly polarized light incident along the *x*-axis passes through the vapor cell, leading to an optical rotation angle *θ*. The linearly polarized light transforms into elliptically polarized light when it passes through the QWP. The rotation angle *θ* is converted into the ellipticity angle *χ* of the elliptically polarized light when the fast axis of the QWP is aligned with the *y*-axis. Thus, the *θ* and *χ* can be determined by separating the RCP and LCP components of the elliptically polarized light with an LCPG, followed by differential detection using two photodetectors (PDs). The experimental findings demonstrated that the method can measure an optical rotation angle as small as 1.48 × 10^−7^ rad. In a dual-beam AM, this method achieves a 13 % improvement in the response coefficient compared to the traditional PBS-based differential detection method. This improvement is attributed to the LCPG’s exceptional circularly polarized extinction ratio (CPER) of up to 3,656 and its remarkable average diffraction efficiency of 99 %. Ultimately, we obtained an average magnetic field sensitivity of 13.8 fT/Hz^1/2^ using this method. In addition, the new method obviates the necessity for an additional vertical optical path, and the diffracted beams are distributed on the same side of the device, which is beneficial to reduce the volume of the detection system. In comparison to traditional polarized optics, the vertical thickness of our device is reduced from millimeters to micrometers, and its geometry can be freely customized to meet different integration requirements. Consequently, our method not only delivers superior performance but also facilitates easier chip-based integration. Our findings pave the way for the advancement of the next generation of high magnetic sensitivity and chip-based AMs, with potential applications in high-spatial-resolution biomagnetic imaging.

## 2 Design and methods

### 2.1 Theory of optical rotation angle detection using the LCPG

Figure 1a shows the principle of optical rotation angle measurement using an LCPG. This measurement process was analyzed using the Jones matrix. Given that the probe light’s polarization direction is along the *y*-axis and the direction of propagation is along the *x*-axis, with the *y*–*z* plane serving as the datum plane of the Jones vector, the Jones vector of the probe light can be represented as follows:

(1) $$E_{0}=A\left[\begin{array}{c} 1\\ 0 \end{array}\right]$$

where *A* represents the amplitude of the probe light. The passage of linearly polarized light through the vapor cell results in an optical rotation angle *θ*, which equates the cell to a Faraday rotation crystal. The Jones matrix of this crystal can be represented as follows:

(2) $$J_{\text{cell}}=\left[\begin{array}{cc} \cos\theta & -\sin\theta \\ \sin\theta & \cos\theta \end{array}\right]$$

The probe light travels through the cell and then through the fast axis along the *y*-axis of the QWP. We can express the Jones vector of the outgoing as follows:

(3) $$\begin{array}{cc} E_{1} & =J_{\text{QW}}\cdot J_{\text{cell}}\cdot E_{0}\\ & =\left[\begin{array}{cc} 1 & 0\\ 0 & -i \end{array}\right]\cdot \left[\begin{array}{cc} \cos\theta & -\sin\theta \\ \sin\theta & \cos\theta \end{array}\right]\cdot A\left[\begin{array}{c} 1\\ 0 \end{array}\right]\\ & =A\left[\begin{array}{c} \cos\theta \\ -i\sin\theta \end{array}\right] \end{array}$$

The outgoing light *E*_1_ is elliptically polarized. The half-length axis *a* = *A*cos*θ* and the half-short axis $b=A\cdot \left|-i\sin\theta \right|=A\sin\theta$ . The ellipticity angle *χ* is defined as follows:

(4) $$\chi =\tan^{-1}\left(\frac{b}{a}\right)=\tan^{-1}\left(\frac{A\sin\theta }{A\cos\theta }\right)=\theta$$

Therefore, a QWP facilitates the conversion of linearly polarized light into elliptically polarized light. The ellipticity angle of the elliptically polarized light and the rotation angle are equal when the fast axis of the QWP is aligned with the polarization direction of the probe light. Thus, detecting the optical rotation angle *θ* is equivalent to detecting the ellipticity angle *χ*.

Subsequently, the LCPG is employed to decompose *E*_1_ into *E*_LCP_ and *E*_RCP_, which ideally have:

(5) $$\begin{array}{ll} E_{1} & =A\left[\begin{array}{c} \cos\theta \\ -i\sin\theta \end{array}\right]\\ & =\frac{A\left(\cos\theta +\sin\theta \right)}{2}\left[\begin{array}{c} 1\\ -i \end{array}\right]+\frac{A\left(\cos\theta -\sin\theta \right)}{2}\left[\begin{array}{c} 1\\ i \end{array}\right]\\ & =E_{\text{LCP}}+E_{\text{RCP}} \end{array}$$

Subsequently, the light intensities detected by PD_1_ and PD_2_ can be expressed as follows:

(6) $$\begin{array}{c} I_{1}=E_{\text{RCP}}\cdot {E_{\text{RCP}}}^{*}=\frac{I_{0}{\left(\cos\theta +\sin\theta \right)}^{2}}{2}\\ I_{2}=E_{\text{LCP}}\cdot {E_{\text{LCP}}}^{*}=\frac{I_{0}{\left(\cos\theta -\sin\theta \right)}^{2}}{2} \end{array}$$

*I*_0_ denotes the light intensity after transmitting through the cell. Given that *θ* is very small, we can assume that *θ* ≪ 1, thereby yielding the following expression:

(7) $$I_{1}-I_{2}=I_{0}\sin\left(2\theta \right)\approx 2I_{0}\theta$$

Given the linear relationship between the optical rotation angle *θ* and the magnetic field *B*_*y*_ (Supplementary Note 1), the resultant AM output signal can be derived as follows:

(8) $$V_{0}=G\left(I_{1}-I_{2}\right)=2GK_{B}I_{0}B_{y}$$

where the output voltage signal of the AM is denoted by *V*_0_, the conversion coefficient between the differential light intensity of the PDs and the output voltage of the AM is represented by *G*, the conversion coefficient between the optical rotation angle *θ* and the magnetic field *B*_*y*_ is represented by *K*_*B*_.

In Eq. (8), the diffraction efficiency *η* and the CPER *σ* ^2^ of the LCPG are not considered. Considering these factors, based on Marius’ law, we obtain:

(9) $$I_{1}^{\prime }-I_{2}^{\prime }=\eta I_{0}\frac{\sigma^{2}-1}{\sigma^{2}+1}\sin\left(2\theta \right)\approx 2\eta I_{0}\frac{\sigma^{2}-1}{\sigma^{2}+1}\theta$$

Equation (9) is also applicable to the traditional differential detection method using PBS and HWP. Here, *η* represents the transmittance of the PBS, which is ideally 1. *σ* ^2^ is the extinction ratio of the PBS, which is ideally infinite. In the context of differential detection in AMs, we are particularly concerned with the performance metrics of beam splitters that directly impact the overall performance of the AM. For this purpose, we define a common quality factor, namely the attenuation coefficient $\mu =\eta \frac{\sigma^{2}-1}{\sigma^{2}+1}$ , applicable to different types of beam splitters, including circular and linear polarization splitters. Therefore, the output signal of the AM is derived as follows:

(10) $$V_{0}^{\prime }=2\mu GK_{B}I_{0}B_{y}$$

When considering the transmittance and extinction ratio of the device, the output signal is only multiplied by the attenuation coefficient compared to the ideal case, with no alteration to its line shape. Moreover, the attenuation coefficient approaches 1 as the transmittance and extinction ratio increase, leading to a greater output signal from the AM. Therefore, the attenuation coefficient is more general than the transmittance or the extinction ratio, especially for performance evaluation and comparison between devices with different materials and different configurations.

### 2.2 Fabrication and performance testing of the LCPG

The LCPG employed in this study was constructed using LC films with a thickness of a few micrometers on a silica substrate (Supplementary Note 2). The fabrication process involved the use of laser direct writing technology combined with photoalignment technology [30], [31]. In particular, the photoalignment layer on the substrate’s surface is achieved by simultaneously altering the polarization state of the laser and the substrate’s position. Subsequently, the photoalignment layer is used to align the LC molecules of the LC layer, leading to a spatial periodic distribution of the internal LC molecules across the surface.

Based on the geometric phase principle, the transmission matrix of the LCPG can be represented as follows [32]:

(11) $$\begin{array}{ll} T & =\cos\left(\frac{\Gamma }{2}\right)\left[\begin{array}{cc} 1 & 0\\ 0 & 1 \end{array}\right]-\frac{i}{2}\sin\left(\frac{\Gamma }{2}\right){\text{e}}^{\text{i}2\phi }\left[\begin{array}{cc} 1 & i\\ i & -1 \end{array}\right]\\ & \;-\frac{i}{2}\sin\left(\frac{\Gamma }{2}\right){\text{e}}^{-\text{i}2\phi }\left[\begin{array}{cc} 1 & -i\\ -i & -1 \end{array}\right] \end{array}$$

where the geometric phase terms are represented by e^i2*ϕ*^ and e^−i2*ϕ*^, the azimuthal angle of the LC molecule that satisfies the period linear variation is as *ϕ*, and Γ represents the birefringent phase delay of the LC layer. The transmission matrix indicates that after passing through the LCPG, the light is divided into three components. The first component is the diffracted light of the 0th order, which retains the same polarization state as the incident light. The LCP and RCP lights are the diffracted light of the positive and negative first orders, respectively, and they carry geometrical phase terms that are conjugate to each other. Therefore, the two diffracted lights will be deflected in opposite directions, with deflection angles satisfying the following equation:

(12) $$\sin\theta_{\pm 1}=\pm \frac{\lambda }{\Lambda }$$

where Λ represents the period of the grating, and the wavelength of the incident light is denoted by *λ*, so the deflection angles can be adjusted by the period of the LCPG. Furthermore, the expression for the diffraction efficiency of the positive and negative first orders is as follows:

(13) $$\eta_{\pm 1}=\frac{1\pm {s^{\prime }}_{3}}{2}\sin^{2}\left(\frac{\Gamma }{2}\right)$$

where $s_{3}^{\prime }$ represents the normalized Stokes parameter. $s_{3}^{\prime }$ is +1 and −1 for the LCP and RCP light, respectively.

The designed LCPG is configured with a period of 9.12 μm, resulting in positive and negative first-order angles of 5° when the wavelength is 795 nm. An optical micrograph of the LCPG obtained under a 100× polarizing microscope is illustrated in Figure 1b (left). The continuously changing brightness signifies a linear change of 180° in the azimuth of the LC molecules within one cycle, corresponding to two light and dark stripes. Considering the generalized Snell’s law of transmission [33], we have constant phase gradient d*φ*/d*x* = 2*π*/Λ with period Λ = 9.12 μm. Then, the phase distribution of the LCPG can be obtained (Figure 1b, right).

**Figure 2:**
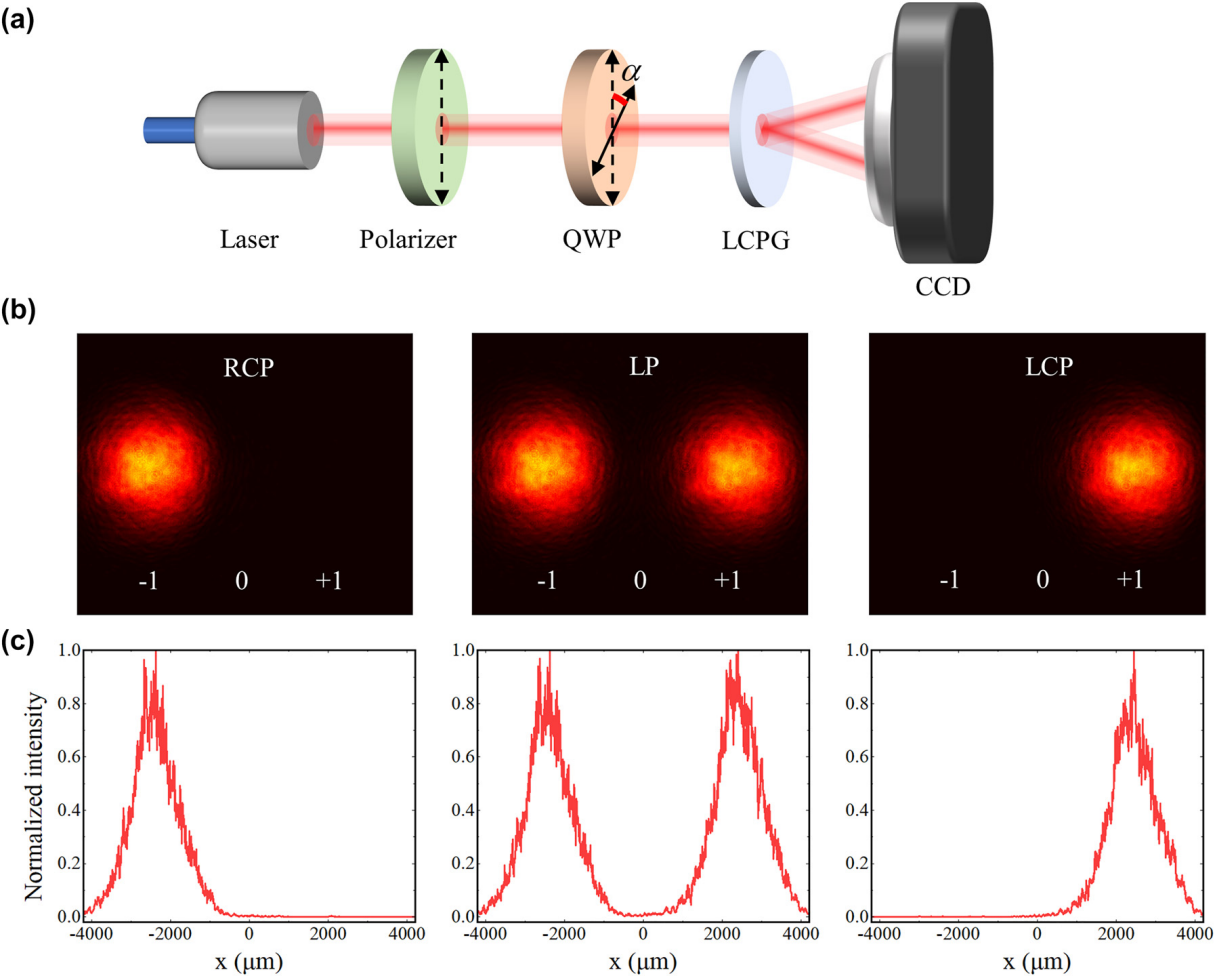
The performance testing of the LCPG. (a) Experimental setup for testing the diffraction and polarization behavior of LCPG. Laser wavelength: 795 nm; QWP: quarter-wave plate; LCPG: liquid crystal polarization grating; CCD: charge-coupled device. (b) Diffraction patterns through the LCPG at RCP, LP, and LCP incidences from left to right. (c) The intensity distribution of the diffracted light at RCP, LP, and LCP incidences from left to right.

To analyze the diffraction and polarization characteristics of the LCPG, the experiment illustrated in Figure 2a was conducted. Outgoing light at a wavelength of 795 nm and a power of 10 mW is generated using a distributed Bragg reflector (DBR) laser, while the polarizer remains fixed in the vertical polarization direction. The fast-axis direction of the QWP (LBTEK QWP25-795A-M) is manually manipulated along the axis, enabling continuous adjustment of *α* within the range of 0 – *π* to achieve RCP, LCP, and linearly polarized light. A camera beam profiler (BC207VIS/M) was used to examine the intensity distribution of the diffracted light and the shape of the spots. The incident light is RCP for *α* = *π*/4, and only a single negative first-order diffraction is produced through the LCPG (Figure 2b and c, the left). The incident light is LCP for *α* = 3*π*/4, and only a single positive first-order diffraction is produced (Figure 2b and c, the right). The incident light is linearly polarized for *α* = *π*/2, and both positive and negative first-order diffractions are produced, which are detected with an intensity ratio of 1:1 (Figure 2b and c, the middle). Subsequently, the impact of varying polarization states and incidence angles on the diffraction efficiency of the LCPG was evaluated (Supplementary Note 3). The findings demonstrate that the diffraction efficiency of the LCPG can be sustained above 98 % under all the aforementioned conditions, with an average diffraction efficiency of 99 %. In addition, the LCPG demonstrates exceptional resilience to the incidence angle, with diffraction efficiency remaining above 97 % at an incidence angle of 20° away from the vertical direction. Consequently, our device demands lower assembly precision in practical applications, facilitating the potential integration of LCPG as a flexible film directly with the vapor cell. We obtained a CPER of 3,656 in a shaded environment by precisely adjusting the QWP (Supplementary Note 3). Superior polarization-selective performance can be obtained using LCPG compared with the extinction ratio range of 1,000–3,000 for conventional bulk PBS.

## 3 Results and analysis

### 3.1 Rotating device design and angular resolution testing

**Figure 3:**
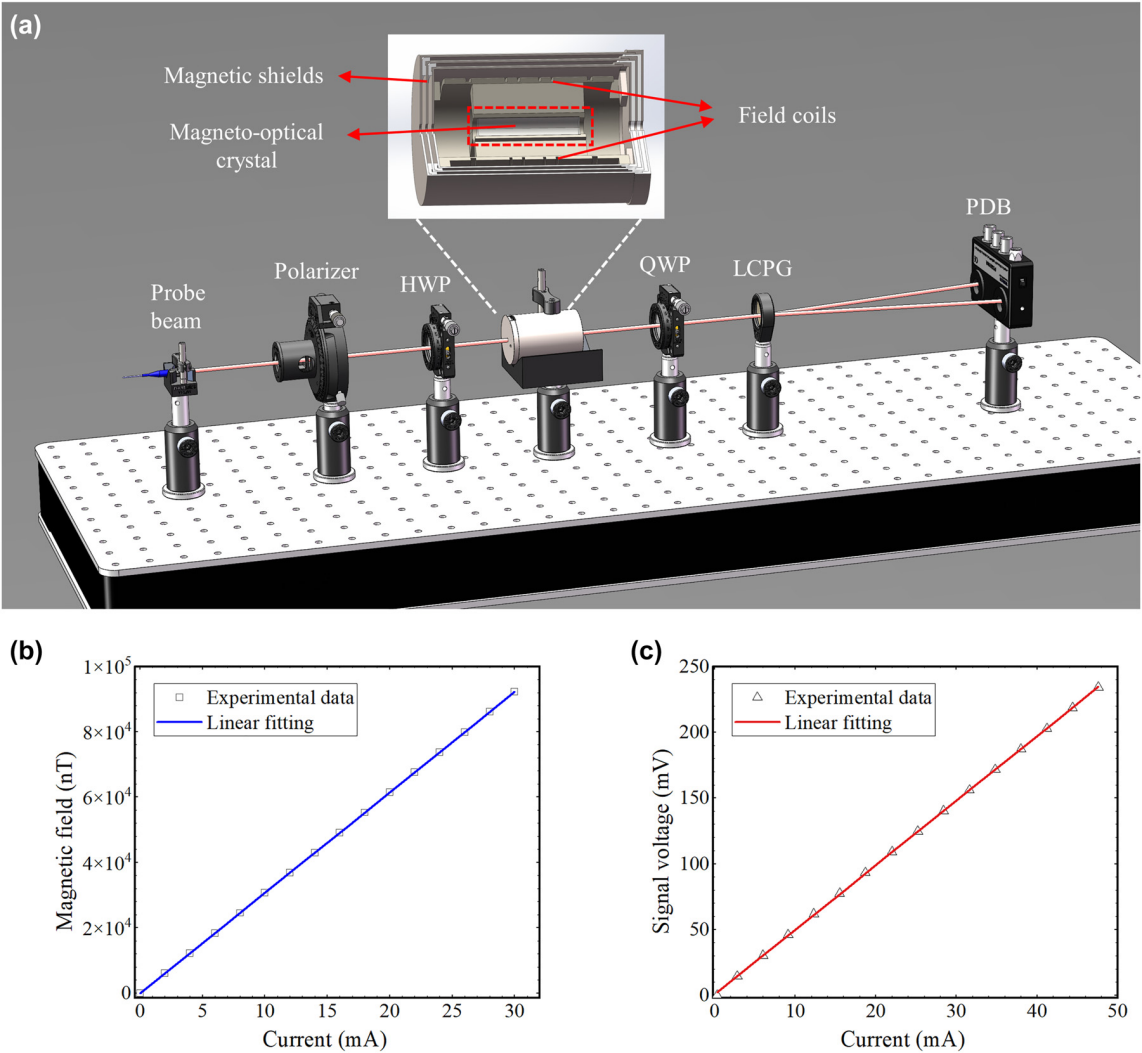
The angular resolution testing of the LCPG-based differential detection method. (a) Experimental setup for measuring the angular resolution using LCPG, the inset shows the structure of a homemade Faraday rotator. PDB: balanced photodetectors. (b) Curve of the solenoid center magnetic field versus the coil driving current. (c) Measurement of output signal voltage versus coil drive current.

To examine the ability of the LCPG-based differential detection method to detect minute angles, a magneto-optical crystal was used to generate stable minute optical rotation angles. Figure 3a shows the experimental device, which comprises a polarizer and an HWP used to produce linearly polarized light with a tunable polarization direction. In addition, we employed a homemade Faraday rotator to produce a tiny optical rotation angle, which was then detected by a differential detection system consisting of the QWP and the LCPG. The inset of Figure 3a shows the structure of the homemade Faraday rotator. We placed a terbium gallium garnet (TGG) magneto-optical crystal at the center of the solenoid and employed a precision current source to apply a driving current to the coil, thereby generating a driving magnetic field. Based on the Faraday effect, when an external magnetic field is present, the rotation angle *θ* of the polarization plane of linearly polarized light after passing through the magneto-optical crystal satisfies the following correlation: *θ* = *VBL*, where *V* represents the Verdet constant of the magneto-optical medium, *B* represents the magnetic field strength, and *L* denotes the effective length of the magneto-optical medium. To isolate the influence of the ambient magnetic field on the TGG, we placed the solenoid inside a small, three-layer magnetically shielded barrel, which was designed with permalloy. Furthermore, a modulated drive current signal is used on the coil to mitigate the influence of low-frequency noise from environmental changes on the detection signal. The modulation current remains correspondingly small when the intended optical rotation angle is extremely small, and the heat produced by the solenoid is insignificant, ensuring that the constant *V* does not fluctuate because of temperature variations during the measurement. Using the aforementioned apparatus negates the effect of external magnetic fields, temperature fluctuations, and other potential sources of interference on the detection signal, thereby facilitating accurate measurement of angular sensitivity.

To generate a small magnetic field, a 120-turn homemade solenoid coil is employed. The correlation between the central magnetic field and the coil driving current was measured using a fluxgate magnetometer with a sensitivity of 0.1 nT. Subsequently, a linear fit was conducted, as illustrated in Figure 3b, yielding a fitting slope of about 3,077.6 nT/mA. This implies that a 1 mA change in the current will change the central magnetic field of the solenoid by 3,077.6 nT. Next, a TGG measuring 20 mm in length and a Wilder coefficient of 75 rad/(T m) at a wavelength of 795 nm is placed at the center of the solenoid. Based on *θ* = *VBL*, *δθ* is approximately 4.55 × 10^−6^ rad/mA when *δB* = 3,077.6 nT. This indicates that a 1 mA change in current will lead to a change in the optical rotation angle of 4.55 × 10^−6^ rad.

The input power of the probe light is approximately 5 mW in a practical experimental system, and a 1 kHz sinusoidal signal source is employed to drive the solenoid and simultaneously act as a reference signal for the lock-in amplifier (Zurich Instruments MFLI). A balanced differential photodetector (PDB210A/M) was used to capture the differential light intensity signal of the positive and negative first orders, which was then demodulated utilizing the lock-in amplifier. The linear relationship between the output signal of the lock-in amplifier and the current is illustrated in Figure 3c. The fitting function can be expressed as *y* = 4.91*x* + 0.73. Therefore, the polarization plane of the linearly polarized light is rotated by 4.55 × 10^−6^ rad and the output signal will increase by 4.91 mV for each 1 mA increase in current. At steady state, the noise voltage associated with the output signal of the lock-in amplifier is approximately 0.16 mV. Therefore, based on the proportionality relationship, the minimum resolvable angle corresponding to the noise voltage can be calculated as 1.48 × 10^−7^ rad. The experimental findings indicate that the approach can measure an optical rotation angle as small as 1.48 × 10^−7^ rad.

### 3.2 Magnetic field measurements

**Figure 4:**
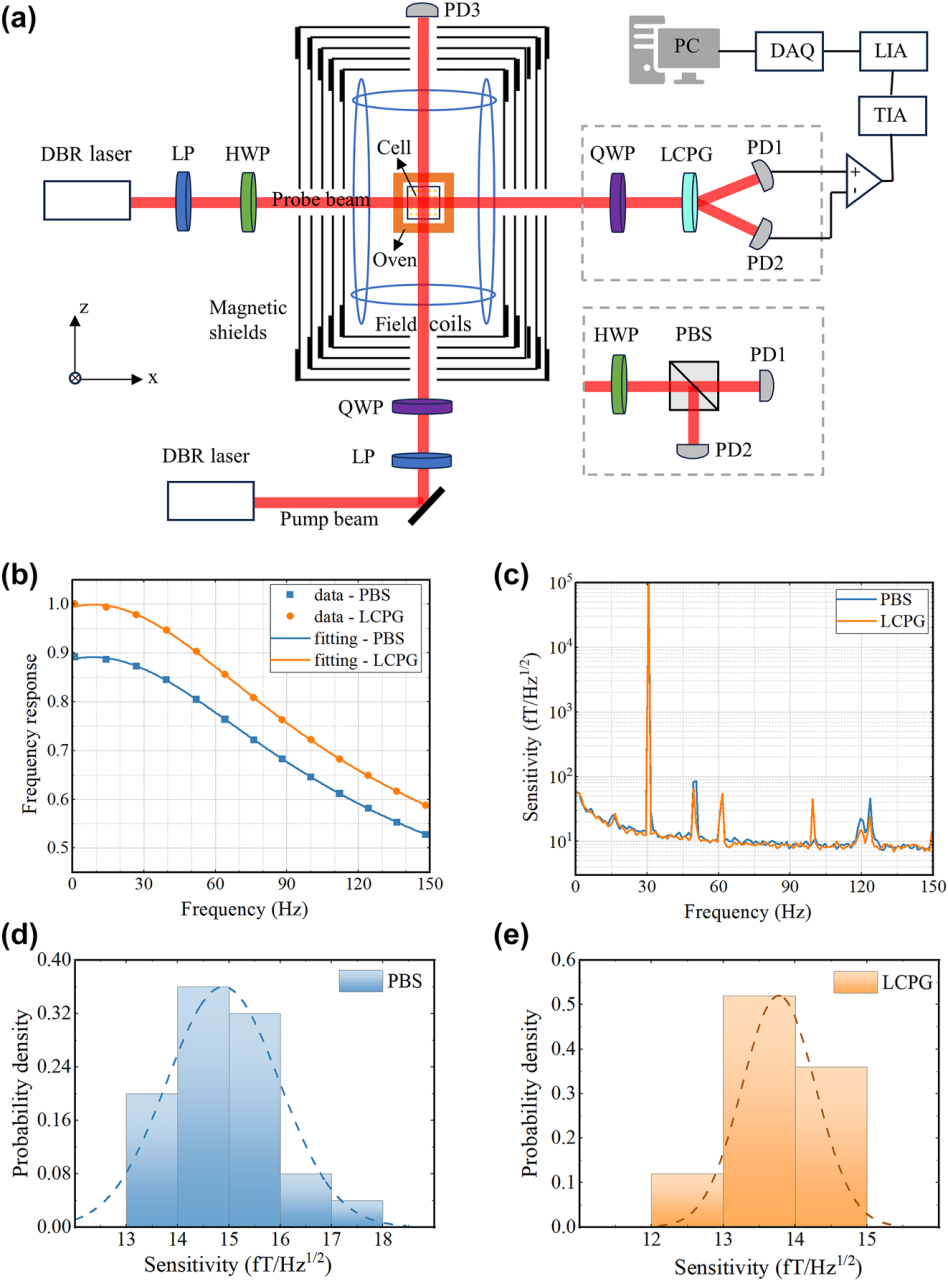
The performance testing of the AM based on PBS and LCPG, respectively. (a) Schematic structure of the magnetometer. LP: linear polarizer; TIA: trans-impedance amplifier; LIA: lock-in amplifier; DAQ: data acquisition. (b) Normalized frequency response curves using PBS and LCPG, respectively. (c) Sensitivity curves using PBS and LCPG, respectively. (d) and (e) Sensitivity normal distribution histograms for PBS and LCPG, the curve in the histogram is the fitted curve corresponding to the test data.

The new method was applied to the AM and compared with the traditional PBS-based detection method. The aim is to assess the impact of this detection method on the response and sensitivity of the magnetometer. Figure 4a shows the experimental setup. The core-sensitive element of the AM is a cubic glass gas cell with an internal dimension of 3 mm. The cell was filled with a small drop of ^87^Rb as the sensitive atom and 600 Torr N_2_, which served as the buffer gas and quenching gas. The cell was then positioned in an oven fitted with two heating films and a PT1000 temperature-measuring resistor. These heating films receive a high-frequency current of 200 kHz, which serves to mitigate the influence of the magnetic field generated by the heating current on the vapor atoms. The cell and oven are located within a magnetic shield consisting of four layers of permalloy and one layer of aluminum. Equipped with triaxial coils, this shield is designed to compensate for residual magnetic field and to apply the calibrated magnetic field. A DBR laser with a central wavelength of 795 nm generates the pump beam. The beam is transformed into circularly polarized light and incident along the *z*-axis into the vapor cell to pump the alkali metal atoms after passing through the linear polarizer (LP), and QWP. Another DBR laser was employed to produce a probe beam, which is detuned by roughly 100 GHz from the center of the 87Rb D1 line. This beam is incident into the cell along its *x*-axis. Subsequently, we employed combinations of a QWP, LCPG, and an HWP, PBS for differential detection to derive the response curves and sensitivities of AM using the two differential detection methods. For PBS-based differential detection, we used a commercial broadband PBS, model GCC-402111. To receive the light intensity signal, two PDs were positioned on the transmitting and reflecting sides of the PBS.

The first step involves evaluating the amplitude-frequency response characteristics. A constant-amplitude alternating magnetic field was applied to the sensitive axis of the AM at a frequency distributed between 1 and 150 Hz. Subsequently, we record the magnitude of the response of the AM to the input field and fit it to the following model:

(14) $$R\left(f\right)=\frac{A}{\sqrt{{\left(f-f_{0}\right)}^{2}+B^{2}}}+C$$

where *A* represents the response coefficient, *f*_0_ denotes the center frequency, *B* represents the AM bandwidth, and *C* denotes the AM bias response.

The amplitude-frequency response was assessed using two different differential detection methods and fitted based on the aforementioned model. For comparison, the frequency responses of the two methods were normalized and the results are shown in Figure 4b. Based on LCPG and PBS differential detection, the fitted amplitude-frequency response curves are as follows:

(15) $$R_{1}\left(f\right)=\frac{66.91}{\sqrt{{\left(f-8.799\right)}^{2}+81.08^{2}}}+0.173$$

(16) $$R_{2}\left(f\right)=\frac{59.15}{\sqrt{{\left(f-8.64\right)}^{2}+81.14^{2}}}+0.162$$

The fitting results indicate that the response coefficients for the two methods are 66.91 and 59.15, respectively, demonstrating a 13 % improvement in the magnetometer response coefficient with our method compared to the traditional PBS-based differential detection method. According to our analysis in Section 2.1, the response coefficient of the AM is positively correlated with the attenuation coefficient of the beam splitter. The diffraction efficiency and CPER of the LCPG are 99 % and 3,656, respectively, corresponding to an attenuation coefficient of 0.989. Theoretically, our device has minimal signal attenuation. However, the angular dependence of the beam splitter can also affect the device’s actual performance. The commercial PBS used (GCC-402111) requires an incident angle within ±2°, meaning even slight misalignments can reduce the magnetometer’s effective response coefficient. In contrast, our device exhibits lower angular dependence, contributing to a higher response coefficient for the magnetometer. Additionally, the magnetometer’s response coefficient is influenced by the transmission rate of the vapor cell and the photoelectric conversion efficiency of the photodetector. Future optimizations in these areas could enhance the system’s response coefficient [34]. Finally, the bandwidth and center frequency of the magnetometers obtained using the two differential approaches are nearly identical. This indicates that the selection of a differential detection device has no impact on the magnetometer’s bandwidth.

The second step involves performing a magnetometer sensitivity test. The procedure was as follows: we applied a calibrated magnetic field signal with an amplitude of 100 pTrms and a frequency of 30.5 Hz to the magnetometer and collected the resulting output voltage signal. The collected data at a sampling frequency of 1,000 Hz are then subjected to a power spectrum analysis for 100 s to obtain the output noise spectrum of the AM with open root sign processing. Subsequently, the noise spectrum is divided by the amplitude-frequency response function, and the effective value of the calibrated magnetic field is used as a reference to derive the normalized noise spectrum of the magnetometer. This enables the sensitivity within the frequency band to be determined based on its noise floor. The sensitivity of the *y*-axis of the magnetometer was assessed using two differential detection methods. Figure 4c shows the resulting normalized sensitivity curves. The sensitivities of the PBS and LCPG were determined by averaging the five points before and after the calibration signal, yielding values of 15.8 fT/Hz^1/2^ and 13.2 fT/Hz^1/2,^ respectively.

To further compare the long-term sensitivity of the two methods, after adopting the calibration magnetic field with a frequency of 30.5 Hz and an amplitude of 100 pTrms, the output signal of the magnetometer is continuously collected for 750 s. The acquired data were then grouped in time intervals of 30 s, and the sensitivities of each group were calculated separately. Figure 4d and e shows the results of the sensitivity data analysis using normal distribution histograms, corresponding to PBS and LCPG, respectively. The ordinate represents the probability density of the corresponding interval, with all probability densities summing to 1. The curves in the histograms represent the corresponding fit curves, from which the average and standard deviation of the long-term sensitivity data can be calculated. The results indicate that the average long-term sensitivities of the two methods are 14.9 fT/Hz^1/2^ and 13.8 fT/Hz^1/2^, respectively. This demonstrates that our device does not introduce additional noise to the AM system. In fact, the sensitivity of AMs is dictated by various types of noise [34], [35], [36]. The enhancement of the response coefficient increases the output signal as well as some noise, while other noise, such as pump light-related technical noise, remain unaffected. Consequently, the overall noise reduction is smaller than the signal enhancement, resulting in improved sensitivity. Since the impact of the beam splitter on magnetometer sensitivity is based on its effect on the response coefficient, we primarily focus on enhancing the beam splitter’s performance and its contribution to a 13 % increase in the response coefficient. In summary, our results indicate that the LCPG-based AM can operate with long-term stability while maintaining high sensitivity.

## 4 Conclusions

This study proposes an efficient and compact PDD method using an LCPG and a liquid crystal QWP to address the issue of large size and low integration of differential detection modules in AM. An average diffraction efficiency of 99 % and a CPER of up to 3,656 at the target operating wavelength of 795 nm were achieved by growing a few micrometer-thick LC material on a silica substrate and then preparing the LCPG using a laser direct-write approach combined with a photo-controlled orientation method. A magneto-optical crystal was used to assess the performance of the LCPG-based differential detection method. The experimental findings demonstrated that a tiny rotation angle detection of 1.48 × 10^−7^ rad is achieved by the detection method. Compared to the PBS-based differential detection method, this method enhances the magnetometer response coefficient by 13 % and finally achieves an average magnetic field sensitivity of 13.8 fT/Hz^1/2^.

In addition, compared with traditional PBS, the vertical thickness of LCPG according to the diffraction principle is reduced from the millimeter scale to the micrometer scale, and it has the potential for integration. As a conceptual illustration, we present a polarization detection device fabricated by machining a QWP and an LCPG on opposite sides of a glass substrate (Supplementary Note 4). The device can be enhanced by integrating it with two chip-based PDs to form an integrated polarization detection unit. As LC technology continues to advance, it is possible to laminate QWP and LCPG as thin films onto the walls of the vapor cell. Their geometries can be optimized and tailored to accommodate various shapes of microfabricated vapor cells. To integrate this technology into chip-enabled AMs, it is crucial to either develop LC materials that can withstand high temperatures or design magnetometers that function effectively at lower temperatures. Both methods are active research areas [37], [38], [39].

## Supplementary Material

Supplementary Material Details
