# Supplementary material for: Ultra-compact and high-precision differential detection method based on liquid crystal polarization grating for miniature atomic magnetometer
Source: Nanophotonics. 2024 Oct 4;13(23):4255–65. doi: 10.1515/nanoph-2024-0309 (PMC11635970; doi:10.1515/nanoph-2024-0309)
Supplement: Supplementary file 1 — Supplementary Material Details [file j_nanoph-2024-0309_suppl_001.docx]

# Supplementary Information for

# Ultra-compact and High-precision Differential Detection Method Based on Liquid Crystal Polarization Grating for Miniature Atomic Magnetometer

Zhibo Cui^1,2,3,4^, Yuhao Wang^1,2,3,4^, Ying Liu^1,2,3,4,5^, Mingke Jin^3^, Jie Sun^1,2,3,4^, Yueyang Zhai^1,2,3,4,5^, Xiangyang Zhou^1,2,3,4,5^, and Zhen Chai^1,2,3,4,5*^

^1^ Key Laboratory of Ultra-Weak Magnetic Field Measurement Technology, Ministry of Education, School of Instrumentation and Optoelectronic Engineering, Beihang University, Beijing 100191, China

^2^ Institute of Large-scale Scientific Facility and Centre for Zero Magnetic Field Science, Beihang University, Beijing 100191, China

^3^ Hangzhou Extremely Weak Magnetic Field Major Science and Technology Infrastructure Research Institute, Hangzhou 310051, China

^4^ Beihang Hangzhou Innovation Institute, Hangzhou 310052, China

^5^ Hefei National Laboratory, Hefei 230088, China

*Corresponding author: zhenchai@buaa.edu.cn

**This file includes:**

Supplementary Notes 1 to 4

Figs. S1 to S3

## Note 1. Theoretical Analysis of Magnetic Field Measurements

In a spin-exchange relaxation-free (SERF) AM using ^87^Rb atoms as the sensitive source, the pump light frequency is adjusted to match the D1 line of ^87^Rb, while the probe light is adjusted to be detuned to the D1 line of the ^87^Rb^[1]^.

The correlation between the optical rotation angle $\theta$ and the projection $P_{x}$ of the atomic spin precession signal on the x-axis can be expressed as follows when linearly polarized light traveling along the x-axis traverses the SERF state vapor cell ^[2]^:

$$\theta=-\frac{\pi}{2}nlr_{e}cP_{x}f_{D1}\mathrm{Im}\left[L\left(\nu-\nu_{D1}\right)\right]$$

where the density of alkali vapor is denoted by $n$, $l$ denotes the effective propagation distance traveled by the beam through the vapor cell, $r_{e}$ represents the classical electron radius, $c$ denotes the speed of light in vacuum, $f_{D1}$ represents the oscillator strength of the D1 transition of ^87^Rb, $\nu_{D1}$ denotes the D1 resonance frequency, and the imaginary part of the complex Lorentzian curve is denoted by $\mathrm{Im}\left[L\left(\nu-\nu_{D1}\right)\right]$.

Consequently, the SERF state vapor cell can be considered macroscopically equivalent to an optically active crystal, and Eq. (1) can be represented using the equivalent Faraday rotation equation:

$$\theta=K_{V}P_{x}l$$

$$K_{V}=-\frac{\pi}{2}nr_{e}cf_{D1}\mathrm{Im}\left[L\left(\nu-\nu_{D1}\right)\right]$$

where $K_{V}$ represents the equivalent rotation coefficient of the vapor cell.

When the probe light is input along the x-axis, the AM exhibits sensitivity to magnetic fields aligned in the y-direction. Therefore, the magnetometer functions within the linear region when the magnetic field $B_{y}$ being measured varies within a small range, represented as follows:

$$P_{x}=\frac{R_{OP}\gamma^{e}}{\left(R_{OP}+R_{rel}\right)^{2}}B_{y}$$

Where $R_{OP}$ is the optical pumping rate, $R_{rel}$ is total relaxation rate, $\gamma^{e}$ is gyromagnetic ratio of electron. Substituting Eq. (S4) into Eq. (S2) yields the following result:

$$\theta=\frac{K_{V}R_{OP}\gamma^{e}l}{\left(R_{OP}+R_{rel}\right)^{2}}B_{y}$$

Therefore, there is a linear correlation between the optical rotation angle $\theta$ and the magnetic field $B_{y}$, and detecting the $B_{y}$ can be achieved by measuring the $\theta$.

## Note 2. The LCPG manufacturing process

The manufacturing process of LCPGs can be divided into the preparation of a glass substrate, the preparation of a photoalignment layer, the performance of laser direct writing, the preparation of a liquid crystal polymer film, and finally, multiple laminations and transfers. Among these steps, the preparation process of the liquid crystal polymer film contains two steps: firstly, the spin-coating of a liquid crystal polymer precursor solution on top of the photoalignment layer, and annealing to form a liquid crystal polymer precursor film. Subsequently, the liquid crystal polymer precursor film is subjected to ultraviolet irradiation, which facilitates the cross-linking of the liquid crystal polymer precursor film, thereby transforming it into a polymer film.


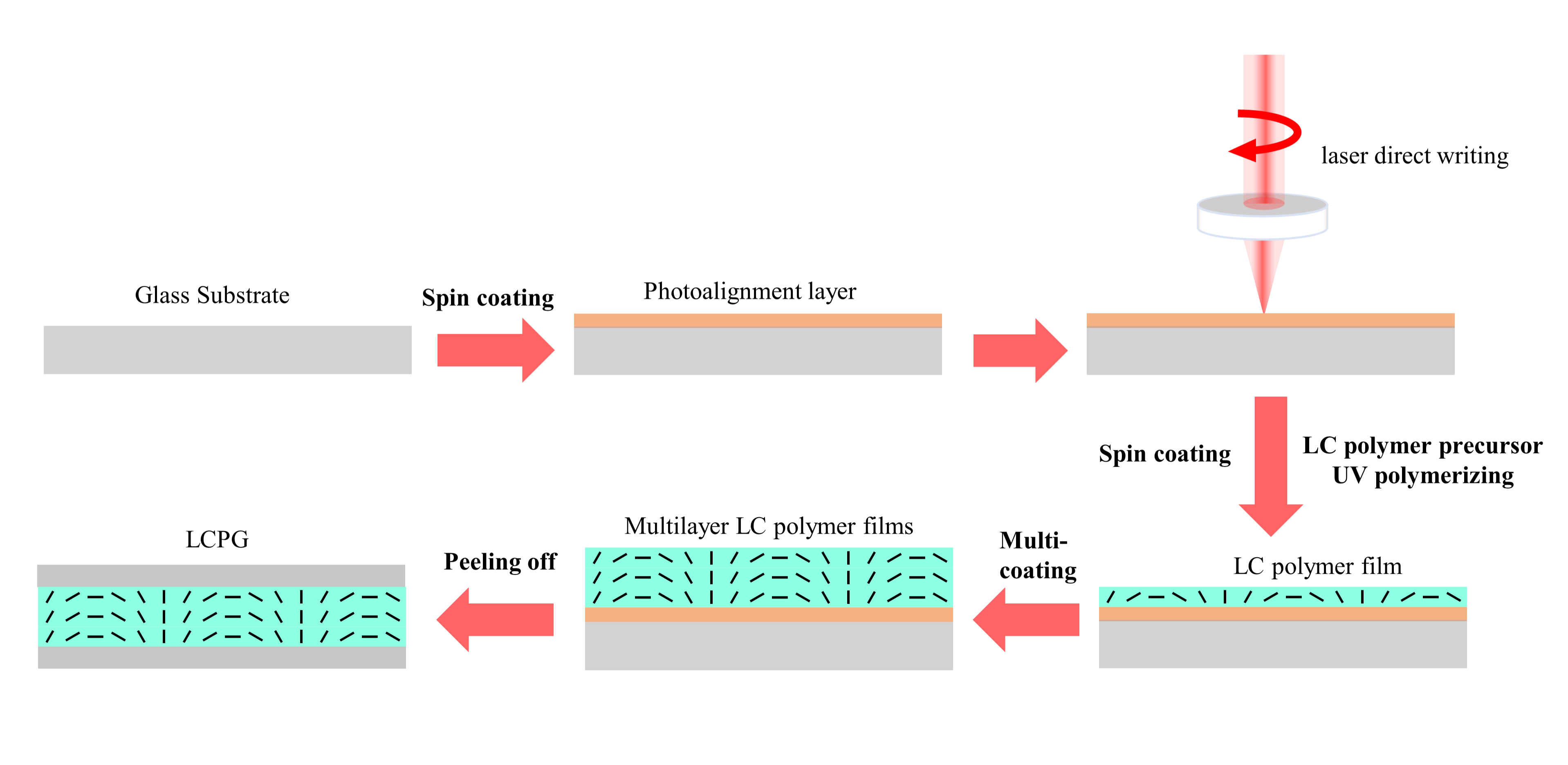


**Fig. S1** The LCPG manufacturing process. LC: Liquid Crystal; UV: Ultraviolet; LCPG: liquid crystal polarization grating.

## Note 3. Diffraction efficiency and extinction ratio of the LCPG

To test the diffraction efficiency and extinction ratio of the LCPG, the experiment illustrated in Fig. S2a was conducted. Outgoing light at a wavelength of 795 nm and a power of about 16 mW is generated using a DBR laser, while the polarizer remains fixed in the vertical polarization direction. The fast-axis direction of the QWP is manually manipulated along the axis, enabling continuous adjustment of $\alpha$ within the range of 0 – π. The diffraction efficiencies (Fig. S2b) and the positive and negative first orders power (Fig. S2e) at various ellipticities of the incident light were determined by continuously rotating the QWP. As shown in Fig. S2b, when the initial linearly polarized light aligns parallel to the grating line direction, the average diffraction efficiency reaches 99.5%, and when the initial linearly polarized light aligns perpendicular to the grating line direction, the average diffraction efficiency reaches 98.5%. Instead of the QWP, the HWP is used and precisely rotated to produce linearly polarized light with different polarization directions. Fig. S2c shows that for all linear polarization angles ranging from 0 to π, the diffraction efficiency remains above 99%. Subsequently, the polarization sensitivity of the LCPG to the incidence angle was assessed. A precision rotating mirror holder is used to adjust the axial direction of the LCPG, thereby ensuring a uniform variation of the incidence angle from −25° to 25°. The LCPG demonstrates exceptional resilience to the incidence angle, with diffraction efficiency remaining above 97% at an incidence angle of 20° from the vertical direction (Fig. S2d).

To assess the polarization-selective performance of the LCPG, the circularly polarized extinction ratio (CPER) is defined as follows: $CPER=I_{-1}^{RCP}/I_{+1}^{RCP}=I_{+1}^{LCP}/I_{-1}^{LCP}$, where $I_{-1}^{RCP}$ represents the power of the positive first-order diffraction under RCP incidence, and so on. We obtained an average CPER of 3656 in a shaded environment by precisely adjusting the QWP so that $\alpha=\pi/4$ or $3\pi/4$ (Fig. S2e).

In this study, we selected a probe light frequency detuned approximately 100 GHz from the center of the 87Rb D1 line, corresponding to a wavelength of about 794.5 nm. In practical applications, the optimal detuning frequency may vary slightly for different vapor cell, typically within a range of 50 GHz, which corresponds to a wavelength variation of 0.01 nm. Theoretically, the manufactured LCPG is intended for specific wavelength detection without concern for wavelength dependence. To ensure rigor, we conducted wavelength sensitivity tests by varying the probe light wavelength. Given that our DBR laser's tunable range is ±2 nm, we tested within this range and found diffraction efficiencies exceeding 99%. Therefore, our device is fully suitable for atomic magnetometers.


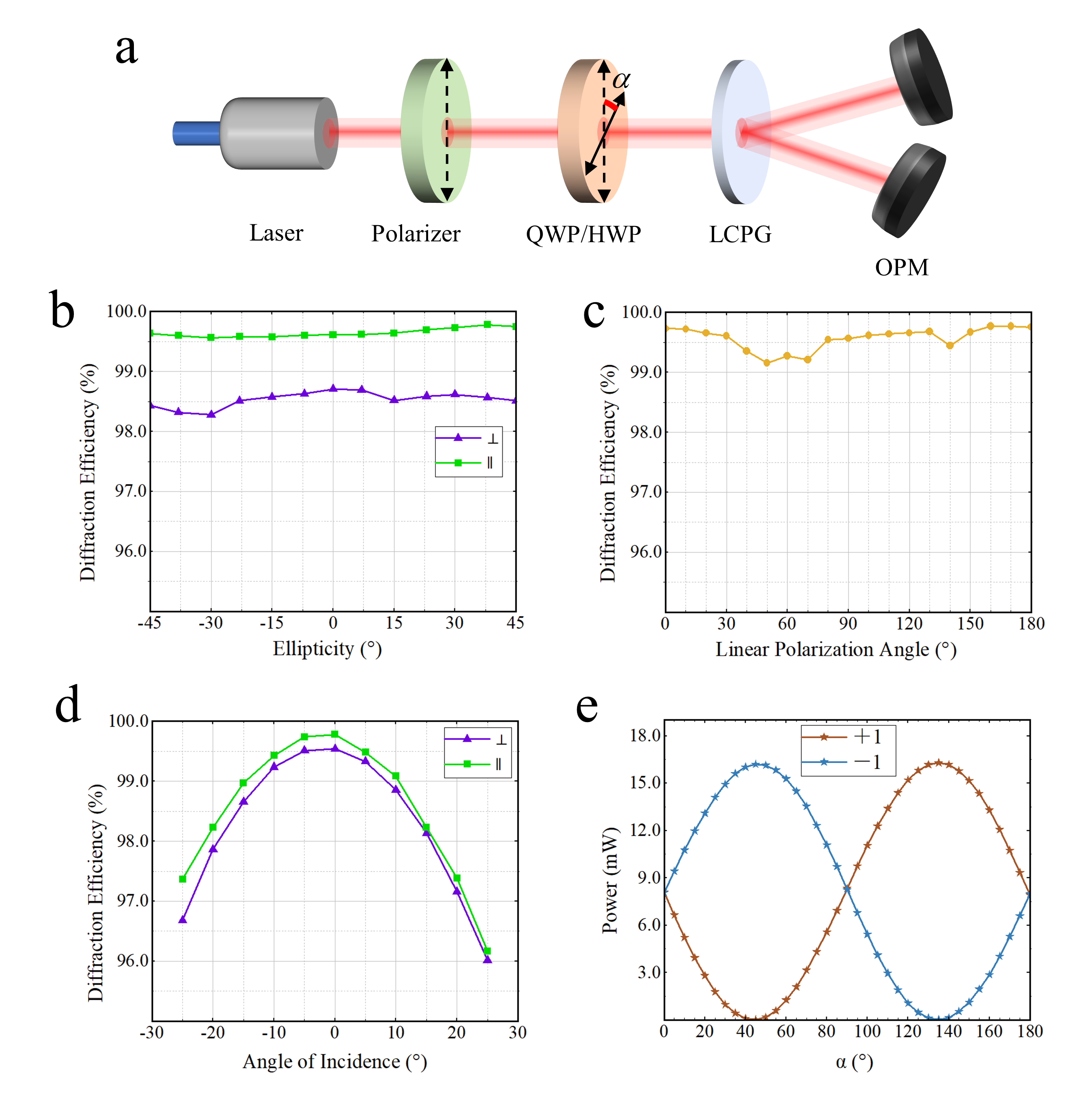


**Fig. S2 a.** Experimental setup for testing the diffraction efficiency and extinction ratio of the LCPG. QWP: quarter-wave plate; HWP: half-wave plate; LCPG: Liquid crystal polarization grating; OPM: optical power meter. b. Diffraction efficiency versus the ellipticity of the incident light. When the ellipticity is 0, it is distinguished according to the polarization direction of the incident light and the grating line direction, which correspond to the triangle and square data in the figure respectively. c. Diffraction efficiency versus the linear polarization angle of the incident light. According to the polarization direction and grid line direction vertical and parallel differentiation, corresponding to triangle and square data in the figure respectively. d. Diffraction efficiency curve with the incidence angle of linearly polarized light. e. The positive and negative first orders power at various ellipticities of the incident light.

## Note 4. An Integrated Polarization Analysis Unit

The figure shows the conceptual diagram of QWP and LCPG integrated on a single substrate.


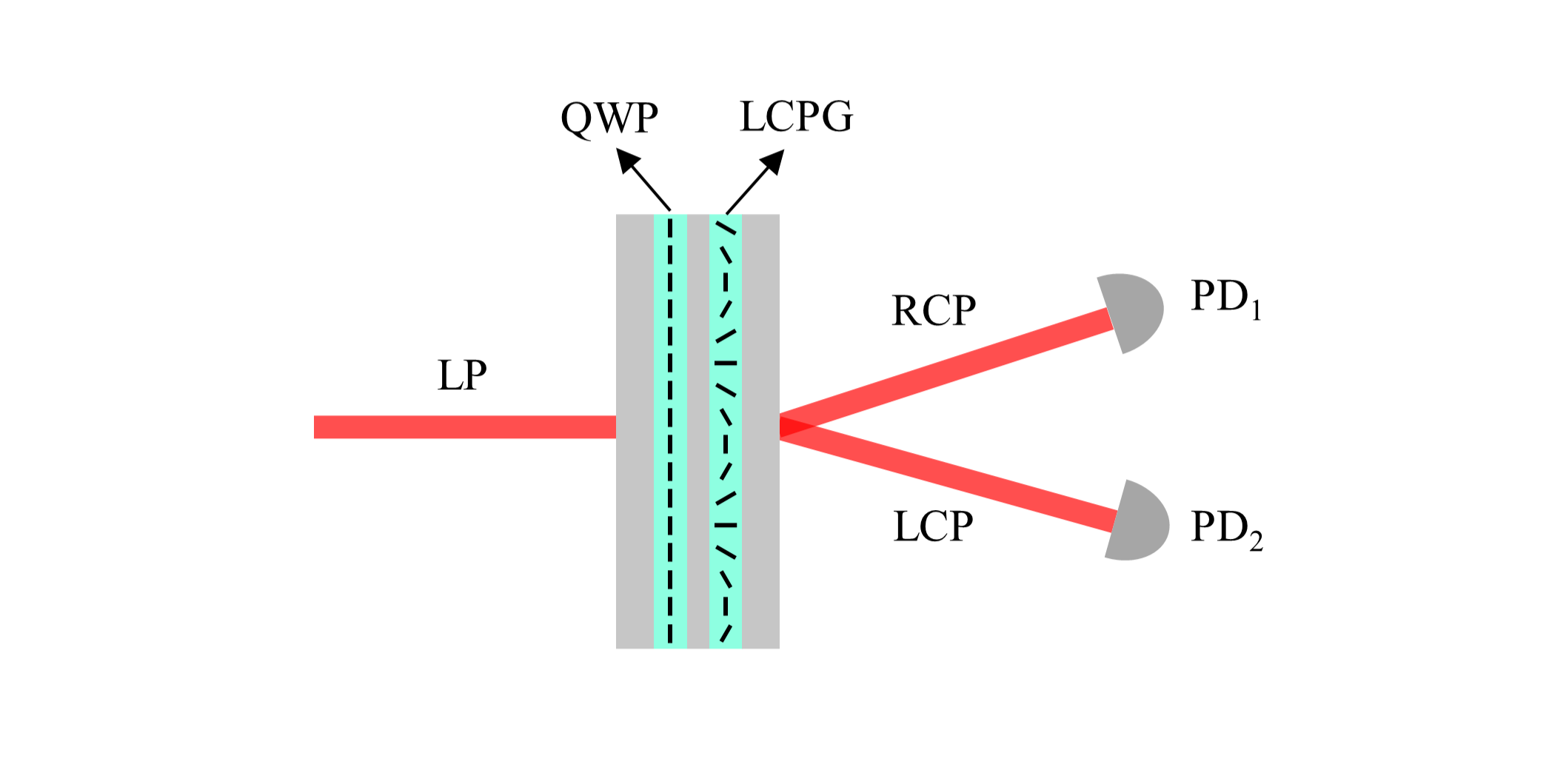


**Fig. S3.** Schematic structure of the integrated polarization analysis unit. QWP: quarter-wave plate; LCPG: Liquid crystal polarization grating; LP: linearly polarized. LCP: left circularly polarized; RCP: right circularly polarized; PD: photodetector.

## References

[1] Shah V., Romalis M. V. Spin-Exchange Relaxation-Free Magnetometry Using Elliptically Polarized Light[J]. Physical Review A, American Physical Society, 2009, 80(1): 013416

[2] Seltzer S. J. Developments in Alkali-Metal Atomic Magnetometry[D].PRINCETON UNIVERSITY, 2008
